# Comparative genetic and epigenetic of the *Sphagneticola trilobata* (L.) Pruski from different regions in China

**DOI:** 10.1186/s12870-023-04277-w

**Published:** 2023-05-30

**Authors:** Yusha Xiao, Xiuqing Chen, Yuhan Yin, Jiening Zheng, Huixian Yi, Liying Song

**Affiliations:** School of Life Sciences, Guangzhou University, Guangzhou, 510006 China

**Keywords:** SSR, MSAP, Sphagneticola trilobata, Epigenetics, DNA methylation, Invasive plants

## Abstract

**Background:**

*Sphagneticola trilobata* (L.) Pruski is a prevalent and widely distributed invasive plant in South China. To investigate the molecular mechanisms underlying its rapid adaptation, we employed DNA methylation-sensitive amplified polymorphism (MSAP) and simple sequence repeat (SSR) analysis to study 60 *S. trilobata* individuals collected from Fuzhou (FZ), Haikou (HK), Jinghong (JH) and Guangzhou (GZ).

**Results:**

In this study, we computed the Shannon diversity index (I) of SSR and MSAP as 0.354 and 0.303, respectively. The UPGMA phylogenetic tree and PCoA analyses showed that MSAP had a better discriminatory power to distinguish populations from different regions. Notably, the GZ population was found to be the most distinct from the other three populations. Moreover, Mantel analysis revealed a significantly higher correlation between epigenetic distance and geographic distance as compared to genetic distance and geographic distance. Consequently, the correlation between epigenetic distance and geographic distance observed to be markedly stronger than that between genetic distance and geographical distance on Mantel analysis.

**Conclusions:**

The *S. trilobata* populations in various regions displayed a high of complementary genetic and epigenetic diversity, which was a key feature contributing to their rapid invasion. Interestingly, the correlation between epigenetics and geographical distance was significantly stronger than that observed for genetics and geographical distance. These findings indicated that the epigenetic mechanism of *S. trilobar* exhibited high plasticity, leading to significant differences in methylation pattern across different populations.

**Supplementary Information:**

The online version contains supplementary material available at 10.1186/s12870-023-04277-w.

## Background

With the globalization of the economy and trade, the rapid expansion in the scope and frequency of human activities has led to the ubiquity of invasive plants, resulting in significant economic losses and ecological damage on a global scale. Genetic and evolutionary processes are believed to be key determinants in the establishment and spread of invasive species. Invasive species must adapt to their new environment in order to survive and reproduce [1], and genetic variation plays an important role in plant adaptation [2]. Therefore, the ability of invasive species to rapidly evolve and adapt to new environmental conditions is a critical factor that determines their success in colonizing new areas and outcompeting native species. According to research, many invasive species exhibit a high level of genetic diversity, which is closely linked to their successful establishment in new environments [3]. For example, the South American weed *Flaveria bidentis* was introduced from Tianjin and quickly spread throughout in northern China. Its ability for sexual reproduction and multiple introductions have led to the maintained of high levels of genetic diversity, which in turn has facilitated local adaptation and invasion [4]. On the other hand, despite having very low genetic diversity, cloned invasive species are still capable of quickly adapting to new environments and becoming successful invaders. Therefore, the genome-level process that translates into phenotypic diversity needs to be re-evaluated. By studying successful plant invaders, we can gain a better understanding of this process.

*Sphagneticola trilobata* (L.) Pruski (synonyme: *Wedelia trilobata*, *S. trilobata* hereafter), belongs to the Asteraceae, a perennial herb native to tropical South America [5]. This plant has been listed as one of the 100 most invasive alien species in the world [6]. It was used widely in landscape greening because of its rapid reproduction, strong survival adaptability, and easy coverage. In the 1970s, *S. trilobata* was introduced to China as a ground cover plant, and *S. trilobata* quickly fled to the wild after settlement, and then developed and spread rapidly in southern China [7].Previous studies have found that *S. trilobata* has the ability to inhibit the growth and development of surrounding species, which poses a significant threat to local biodiversity and causes the imbalance of local ecological diversity. This is due to its rapid coverage of occupied open spaces through allelopathic therapy and asexual reproduction [8, 9]. Moreover, *S. trilobata* can invade other remote areas through water flow or human activities as its stem can directly develop into a complete plant [10, 11]. Currently, it is distributed in Guangdong, Guangxi, Fujian, Hainan, Yunnan and other provinces, mainly in parks, scenic spots, riversides, roadsides, fields, etc.

The extract from *S. trilobata* leaves has been shown to inhibit the germination of seeds and the growth of seedlings from both competitors and its own seeds, resulting in a high number of seed sterilizations and few seedlings in wild populations [12]. Consequently, *S. trilobata* primarily encroaches on new habitats through asexual clonal reproduction. In general, clonal reproduction typically leads to reduced gene flow between individuals within a species and a low level of genetic diversity, which is not conducive to adapting to changing environmental conditions. However, this was contradictory to the fact that *S. trilobata* was highly adaptable to diverse environments. Some researches have shown that epigenetics can provide clonal plants with more adaptive strategies. Invasive species tend to have higher phenotypic plasticity than non-invasive or native species, and even though invasive species populations in invasive areas were able to evolve greater plasticity than native populations [13]. For instance, the invasive plant *Alternanthera philoxeroides*, which predominated in clonal reproduction, responded rapidly to environmental stresses mainly through epigenetic variation [14]. Thus far, the molecular mechanisms of *S. trilobata*'s environmental adaptations has been poorly reported. There was still unclearly about how *S. trilobata* itself adapted quickly in a short term. Therefore, it was hypothesized that *S. trilobata* might adapt quickly to the stresses of different environments in a similar manner.

Epigenetics provides clonal plants with more adaptive strategies. It is one of the best examples of how cloning can replace the slow mechanism of natural selection. Epigenetics depends to varying degrees on asexual reproduction and preserves or reverses gene ability to buffer plants against current challenges and future rapid environmental changes [15]. In addition, epigenetic modifications are genetically stable across generations [16], meaning that more favorable phenotypic variations can be transferred to future generations. Epigenetics refers to changes in gene expression levels based on covalent modifications on DNA or histones, rather than changes in the gene sequence, which are hereditary and inheritably reversible. Plants use epigenetic modifications of the genome to change the gene expression of individuals with the same genotype in response to changes in environmental conditions through phenotypic plasticity. The evolution of epigenetics may be driven by natural selection, indicating that in natural populations, epigenetics and genetic variation are two intertwined rather than independent evolutionary factors [17–19].

DNA methylation, RNA interference, and histone modification are widely recognized as essential components of epigenetic regulation. Among them, cytosine methylation stands out as the most important epigenetic regulation strategy and plays a central role in the epigenetic control of gene expression [20]. Furthermore, the hypothesis of epigenetic characteristics associated with invasion was largely based on that changes in DNA methylation were involved in gene expression regulation, particularly that methylation of gene promoters is associated with gene silencing [21]. It is worth noting that DNA methylation is one of the most common epigenetic modifications. On the contrary, in the contex of exploring genetic diversity research, due to the advantages of abundant multiple alleles, high polymorphism and dominance, simple sequence repeat (SSR) is a powerful tool for studying genetic diversity [22].

Since DNA methylation-sensitive amplified polymorphism (MSAP) does not require sequencing of a reference genome, it can be directly used to assess the methylation status of randomly distributed genomic cytosines. This method has the advantage of being easy to operate and inexpensive, and is currently the most widely used method for detecting cytosine methylation, which often occurs at the CG site in the promoter region of the DNA sequence [23]. MSAP is a method that can be used to directly assess the methylation status of randomly distributed genomic cytosines. Since it does not require sequencing of a reference genome, this method is widely used in the field of analytical epigenetics for detecting cytosine methylation changes in the ecological environment [24].

Previous studies on the invasion mechanism of *S. trilobata* have mainly focused on examining changes in phenotype or physiological indices in response to different environmental factors. However, there are few explorations based on genetics and epigenetics [25]. For better understanding the process of rapid adaptation of *S. trilobata, t*his study explored the genetic and epigenetic structures and differences among populations of *S. trilobata* under different habitats by using molecular markers SSR and MSAP.

## Materials and methods

### Study species

*Sphagneticola trilobata* (L.) Pruski is a common invasive plant widely distributed in South China. It has been recorded in Guangdong, Guangxi, Fujian, Hainan and Yunnan province. It can adpapt to highly heterogeneous environments, such as parks, scenic spots, riversides, roadsides, fields, etc. *S. trilobata,* triangular-shaped leaves with toothed edges that are 5–10cm long and 2–5cm wide. The leaves are arranged alternately on the stem and are slightly hairy on both surfaces. The plant produces yellow daisy-like flowers with a diameter of approximately 2–3 cm, which bloom throughout the year.such as its triangular-shaped leaves and hairy surfaces. The plant's growth habit and morphology also vary depending on its environment, with plants growing in full sunlight typically being more compact and bushy than those growing in shade [10, 26].

### Collection of meteorological data and materials

The current *S. trilobata* distribution records in China were obtained from literature reports and previous data collected by the laboratory. Maxent software and ArcGIS were utilized to predict contemporary suitable areas, and samples were gathered from highly suitable regions. As this species was introduced in the 1970s and has undergone a certain period of evolution, reference data from contemporary climate were chosen as the environmental factor data. The environmental data were collected from China Meteorological Administration.s website (http://www.cma.gov.cn/). In this study, a total of 120 samples were collected solely from highly suitable areas in four different regions: Guangzhou, Guangdong Province (GZ), Haikou, Hainan Province (HK), Fuzhou, Fujian Province (FZ), and Jinghong, Yunnan Province (JH), respectively. Thirty samples were collected from each region (Table 1). The detailed sampling method we applied was as follows: 1) Three quadrats were randomly selected from each region, ensuring that each quadrat was more than 2 km apart (this was sample determined by the specific distribution location of *S. trilobata* in the area); 2) Ten samples were randomly selected from each quadrat and ensure each plant is more than 50 m apart; 3) Harvest the mature leaves after the third pair of leaves on the plant; 4) A total of 120 samples were collected from four populations, and the mature leaves were dried and numbered using silica gel from each region. DNA was extracted from each sample and stored at − 20 °C. Total genomic DNA extraction was extracted using the plant genome DNA extraction kit (Beijing Tiangen).

**Table1:** The information and environmental factors in sampling sites of *Sphagneticola trilobata* from different regions in China

Population	FZ	JH	HK	GZ
Longitude	119°18′	101°14′	101°15′	113°27′
Latitude	26°4′	21°55′	19°57′	23°13′
Altitude(m)	84	574.29	46.68	18
Average annual temperature(℃)	19.68	18.15	24.13	22.06
Highest temperature(℃)	38.21	32.95	37.31	36.78
Lowest temperature(℃)	1.32	2.03	7.67	2.63
Average annual sunshine hours(h)	1654.43	1994.19	1982.64	1686.15
Average annual precipitation(mm)	1374.36	1442.25	1689.7	1766.05
Maximum daily precipitation(mm)	103.81	82.29	167.64	126.53
Average annual relative humidity(%)	74.58	77.8	83.5	77.42

The data is the average from 1952 to 2016

### SSR method

Based on the transcriptome data, six pairs of SSR primers were selected for capillary electrophoresis. The volume of Polymerase Chain Reaction (PCR) system was 10 μL, consisting of 1 μL of DNA template, 5 μL of 2 × Taq PCR Master Mix, 0.5 μL of upstream primers (the 5’ end of the upstream primer was labeled with phosphorescent dye during fluorescence PCR), 0.5 μL of downstream primers and 3 μL of ddH_2_O. The PCR conditions were set as follows: 95 ℃, 3 min; 34 cycles of (95 ℃, 30 s; The annealing temperature is the annealing temperature of each primer, 30 s; 72 ℃, 60 s); and a final extension at 72 ℃ for 5 min, and finally stored at -20 °C. In the end, PCR products were separated by 6% non-denaturing polyacrylamide gel electrophoresis [27].

### MSAP method

Using EcoRI and two restriction sites 5′-CCGG-3′, the restriction endonucleases MspI and HpaII with different sensitivity to methylation were cut at 37 °C for 12 h. The resulting DNA digested products were connected and amplified by PCR. After 2% agarose gel detection, 8 pairs of primers were selected for PCR selective amplification. The selective PCR products were detected with 6% polypropylene gel, and then the color was observed by silver staining (detailed steps can be found in the supplementary materials). There were four cytosine methylation patterns in DNA (Table 2). Type I: sites with the presence of bands in both EcoRI/HpaII and EcoRI/MspI, namely (1.1), it represents CCGG site was unmethylated. Type II: bands present in EcoRI/HpaII, but absent in EcoRI/MspI, namely (1.0), indicating that the external cytosine site of only one DNA chain was methylated, which was also known as the Hemi-methylation of CCGG site. Type III: bands present in EcoRI/HpaII, but absent in EcoRI/MspI, namely (0.1), stands for the internal cytosine of the double DNA chain was methylated, which was also known as the complete methylation of CCGG site. Type IV: bands has not present in EcoRI/HpaII and EcoRI/MspI, namely (0.0). The markers for type IV mode (0.0) were treated as hypermethylated sites in this study followed [28–30].

**Table 2:** DNA methylation band types

The type of bands H M	DNA methylation status	DNA methylation patterns
1 1	Non-methylation	I
1 0	Hemi-methylation	II
0 1	Full-methylation	III
0 0	Hypermethylation	IV

H = EcoRI  +  HpaII; M=EcoRI + MspI

### Data analysis

The SSR bands of *S. trilobata* were quantified by numbers, where “1” denotes the presence of a band and “0” denotes the presence of no band. Similarly, the MSAP bands were counted by numbers, where “1” indicating DNA methylation, and “0” indicating non-methylation. The genetic diversity was calculated using Popgene software, and the phylogenetic trees of SSR with MSAP were constructed using MEGA7. NTSYS software was used to conduct evolutionary tree analysis; PCoA analysis was performed on the the resulting data. Furthermore, GenAlex software was used for mantel test analysis and AMOVA analysis. The R package (4.1.2) and Python (3.10.5) were utilized for data analysis and figure drawing.

## Result and analysis

### Genetic diversity and epigenetic genetic diversity analysis

The numbers of observed alleles (Na) in SSR ranged from 1.656 to 1.750, while the number of effective alleles (Ne) range from 1.319 to 1.530. The average values of Na and Ne were 1.703 and 1.406, respectively. In MSAP, Na ranges from 1.603 to 2.000, while the Ne ranges from 1.148 to 1.344, with the average values of 1.798 and 1.275, respectively. The average values of gene diversity (H) and the Shannon diversity index (I) of both SSR and MSAP were 0.236, 0.184 and 0.354, 0.303, respectively, with little variance (Table 3). In summary, compared with other populations, the genetic diversity of the GZ population was the highest, with slightly lower epigenetic diversity than that of the FZ population, while the genetic and epigenetic diversities of the HK population were significantly lower than those of the other three populations. In terms of genetic distance (Table 4), HK and FZ were the closest, while GZ and JH were the farthest. The epigenetics of JH and FZ were the closest, while the epigenetics of JH and HK were the farthest.

**Table 3:** Genetic and epigenetic diversity analysis on the four *Sphagneticola trilobata* populations from different invasive regions in china

**Population**		**PIC**	**Na**	**Ne**	**H**	**I**
FZ	genetic	75.00%	1.750	1.375	0.228	0.349
	epigenetic	83.47%	1.835	1.344	0.224	0.357
HK	genetic	65.62%	1.656	1.319	0.193	0.297
	epigenetic	60.33%	1.603	1.148	0.101	0.174
JH	genetic	68.75%	1.688	1.399	0.232	0.348
	epigenetic	75.21%	1.752	1.327	0.207	0.326
GZ	genetic	71.88%	1.719	1.530	0.291	0.421
	epigenetic	100.00%	2.000	1.279	0.206	0.353
SSR Mean		70.30%	1.703	1.406	0.236	0.354
SSR Species level			1.969	1.528	0.313	0.472
MSAP Mean		79.75%	1.798	1.275	0.184	0.303
MSAP Species level			2.000	1.457	0.281	0.440

**Table 4:** The distance of genetic and the distance of epigenetic genetic between different *Sphagneticola trilobata* populations in china

	**genetic distance**					**epigenetic distance**			
**Population**	**FZ**	**HK**	**JH**	**GZ**	**Population**	**FZ**	**HK**	**JH**	**GZ**
FZ	-	0.875	0.870	0.134	FZ	-	0.906	0.962	0.837
HK	0.134	-	0.788	0.142	HK	0.099	-	0.018	0.647
JH	0.139	0.238	-	0.078	JH	0.039	0.983	-	0.722
GZ	0.874	0.868	0.925	-	GZ	0.178	0.436	0.325	-

Nei’s genetic/epigenetic distance (left bottom) and genetic/epigenetic identity (top right)

### Cluster analysis of genetics and epigenetics of different populations

**Fig. 1:**
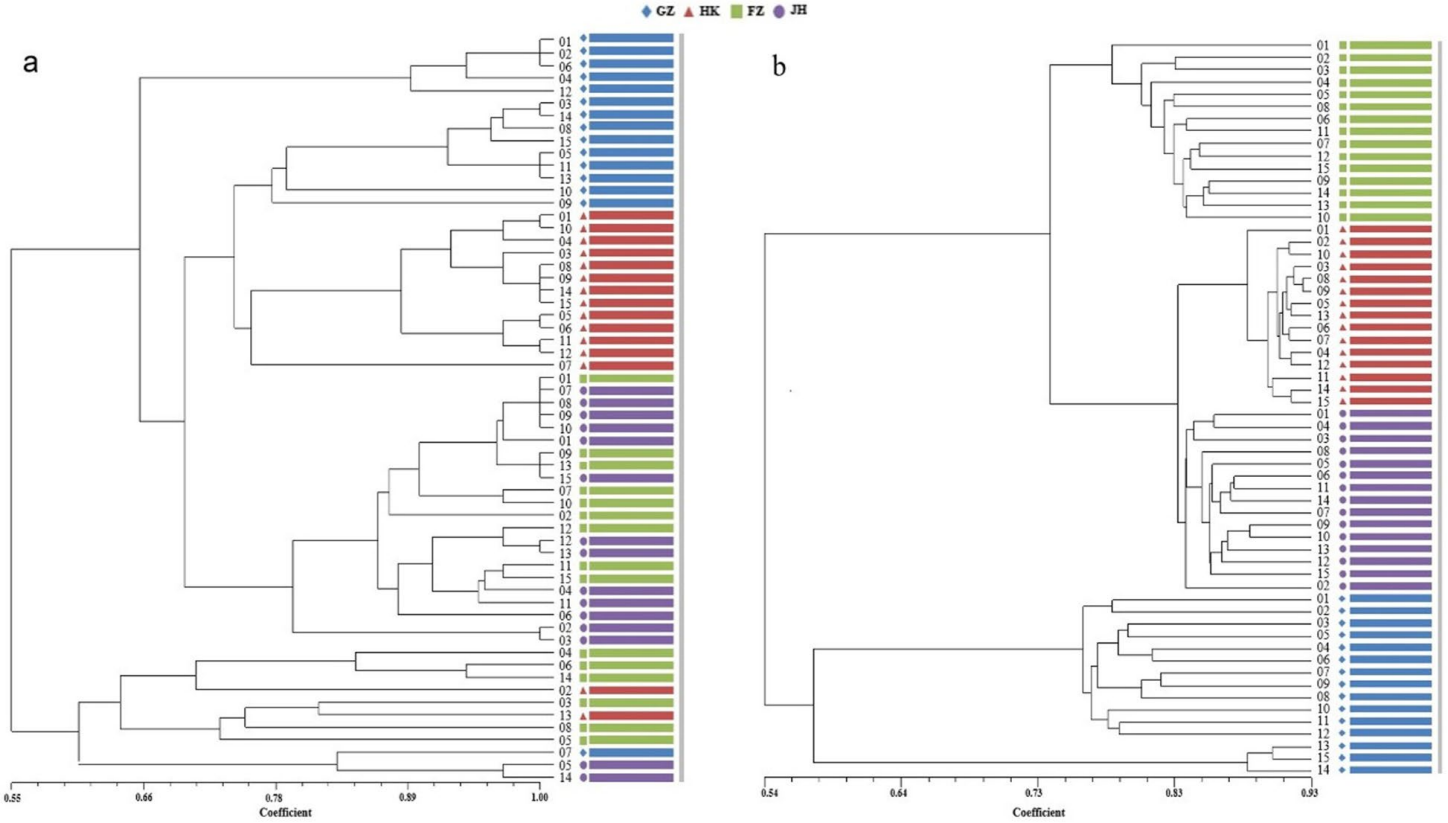
UPGMA clustering analysis reveals differences between four populations of *Sphagneticola trilobata* in China based on SSR (**a**) and MSAP (**b**) analysis

**Fig. 2:**
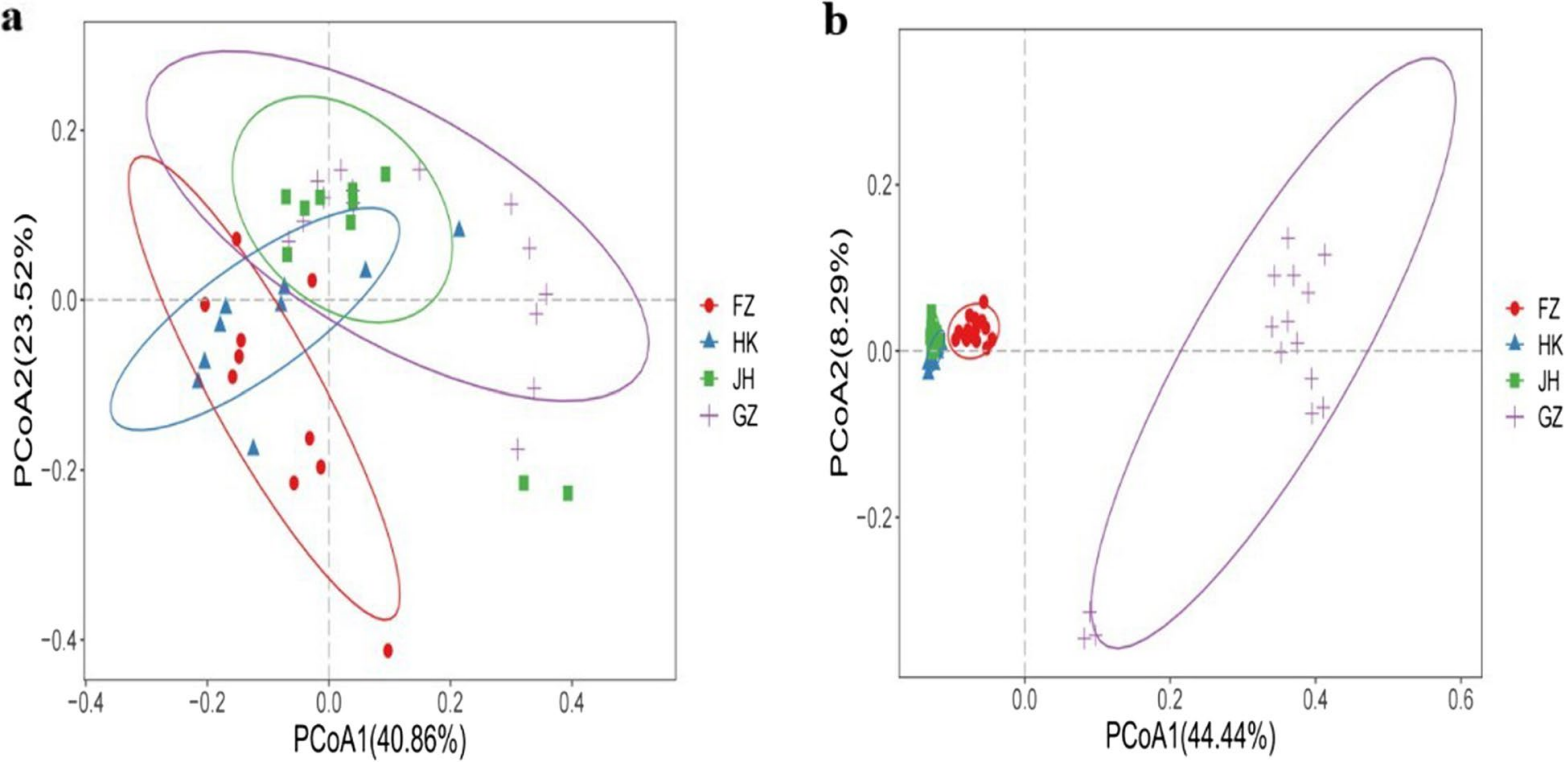
Principal Coordinate Analysis (PCoA) showing the divergence in four *Sphagneticola trilobata* populations in China based on SSR (**a**) and MSAP (**b**) data

In Fig. 1, it can be observed that most samples from HK and GZ regions were clearly clustered into independent branches, while samples from JH and FZ regions were partially intertwined. The cluster of MSAP molecular markers displays a greater degree of independence compared to the SSR cluster in each region. The Principal Coordinate Analysis (PCoA) results reveal that the JH and GZ populations were closely related based on SSR, but they were separated from the FZ population (Fig. 2a). The PCoA findings was consistent with the results from UPGMA clustering. Based on MSAP, FZ, HK and JH populations were close, the second axis distinguishing these three populations from the GZ population (Fig. 2b).

### Correlation analysis of genetic and epigenetic distance and geographic distance for all populations

**Fig. 3:**
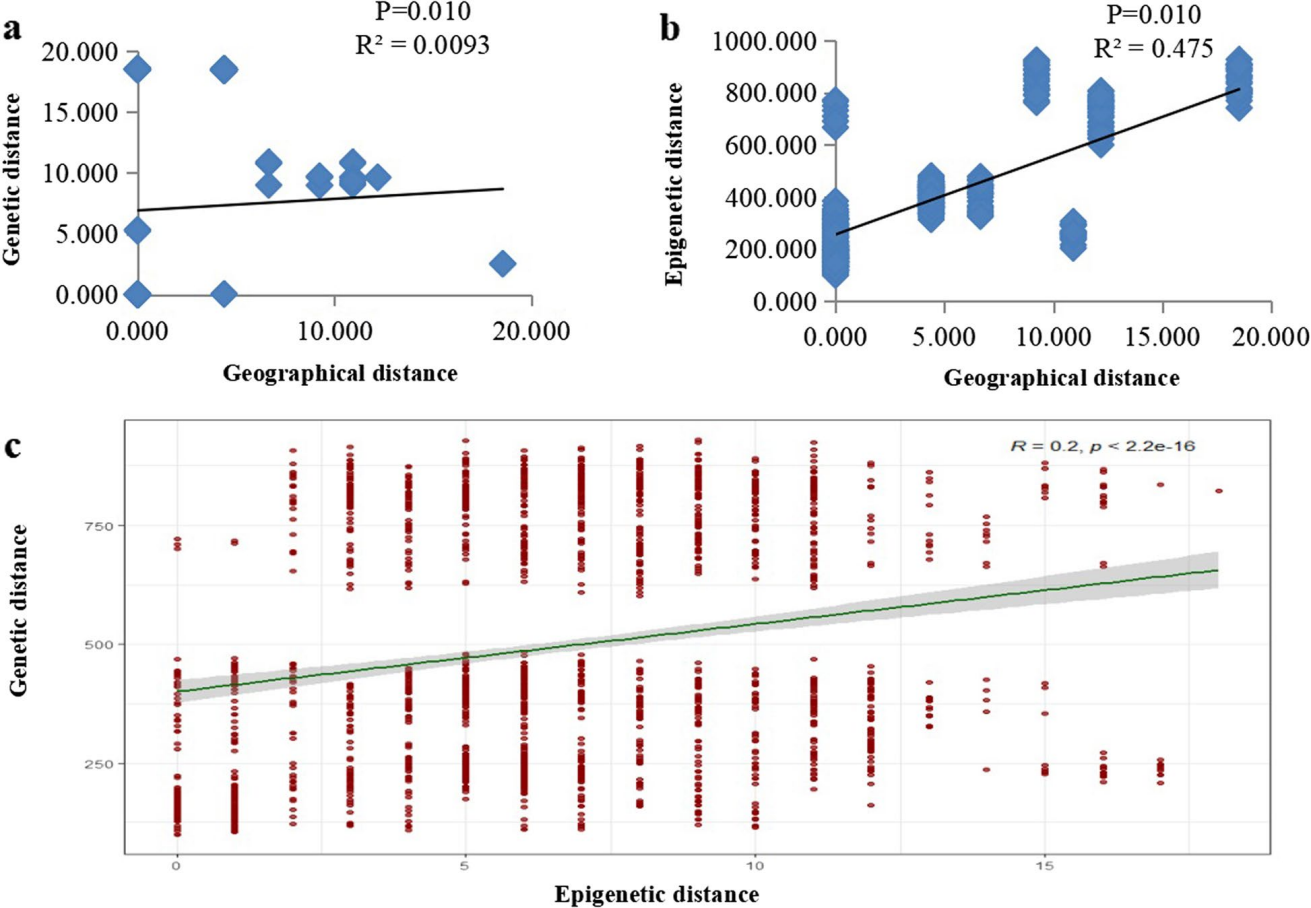
The correlation between the genetic distance (**a**) and epigenetic distance (**b**) of all populations of Sphagneticola trilobata in China and geographical changes, as well as the correlation between the calculated genetic distance and epigenetic distance (**c**)

Mantel analysis of the relationship between geographical distance and genetic or epigenetic distance indicated that the R values were 0.0093 and 0.475, respectively, both with the 0.01 P values (Fig. 3a and b). In addition, the Mantel analysis examining the relationship between genetic and epigenetic distances yielded an R value of 0.2 (Fig. 3c), suggesting a stronger correlation in contrast to genetic distance.

### AMOVA analysis of SSR and MSAP

AMOVA analysis (Table 5) revealed that the epigenetic fixation index (Fst) of *S. trilobata* in different regions was 0.475, which was higher than the genetic fixation index (0.277). This suggested that the differentiation among populations was mainly attributed to epigenetics. Genetic and epigenetic gene flow (Nm) were 0.652 and 0.276, respectively, which were less than 1, indicating that gene flow among *S. trilobata* populations in different regions was limited by geographical distance.

**Table 5:** AMOVA analysis of genetic and epigenetic genetic between different *Sphagneticola trilobata* populations in China

**Source**		**df**	**SS**	**MS**	**Est. Var**	**%**	**Fst**	**Nm**	**P value**
SSR	Among Pops	3	141.233	47.078	1.529	18%	0.277	0.652	0.001
	Among Indiv	56	67.800	1.211	0.000	0%			
	Within Indiv	60	406.000	6.767	6.767	82%			
	Total	119	615.033		8.296	100%			
MSAP	Among Pops	3	3806.350	1268.783	40.222	47%	0.475	0.276	0.001
	Among Indiv	56	3478.133	62.110	17.646	21%			
	Within Indiv	60	1609.000	26.817	26.817	32%			
	Total	119	8893.483		84.686	100%			

### Mode structure of epigenetic regions

**Fig. 4:**
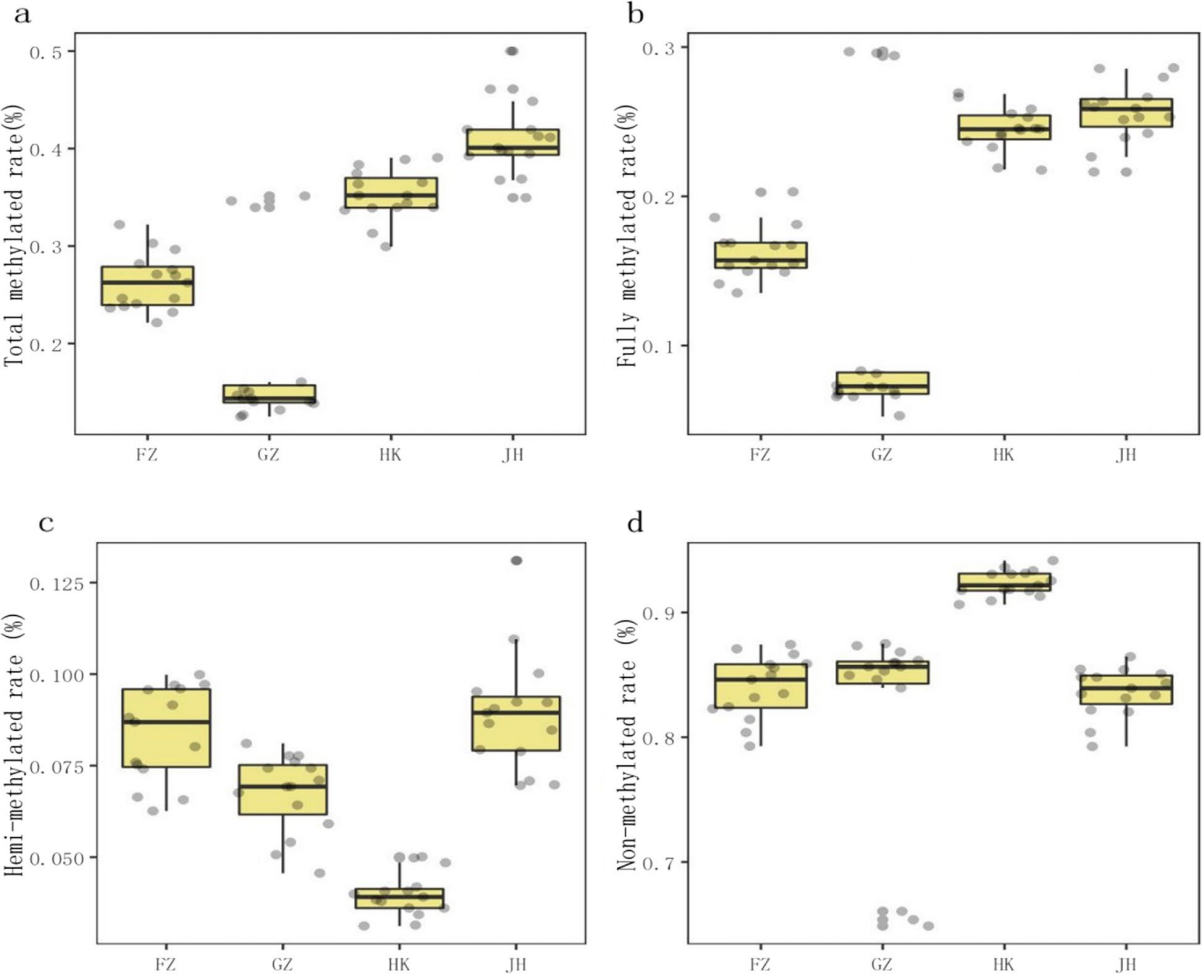
Comparation of *Sphagneticola trilobata* methylation status from different invasive areas in China Note: Each gray dot represents a sample collected from each region for MSAP detection, with a total of 15 samples collected from each region; Total methylated bands =  I + II + III + IV; Full-methylated bands = III + IV; Total methylated rate/% = [(II + III + IV)/(I + II + III + IV)] × 100%; Fully methylated rate(%) = [(III + IV)/(I + II + III + IV)] × 100%; Hemi-methylated rate (%) = [(II)/(I + II + III + IV)] × 100%; Non-methylated rate (%) = [(I)/(I + II + III + IV)] × 100%

The total methylated rates in the four populations (Fig. 4) were 23.82%, 18.25%, 32.92% and 27.55%, respectively; the fully methylated rates were 16.25%, 11.51%, 25.75% and 24.43%, respectively; the hemi-methylation rates were 7.57%, 6.75%, 7.16% and 3.12%, respectively; moreover, the non-methylated rates were 76.18%, 81.75%, 67.08% and 72.45%, respectively.

## Discussion

### Differences in genetic and epigenetic composition in *S. trilobata* rapid invasion

In this study, the Shannon Diversity Indexes (I) of genetic diversity and epigenetic diversity in *S. trilobata* were 0.472 and 0.440, respectively. In *Alternanthera philoxeroides*, the Shannon Diversity Indexes (I) of genetic diversity and epigenetic diversity were 0.002 and 0.046, respectively [14].These results indicated *S. trilobata* has relatively higher genetic and epigenetic diversity. The Shannon Diversity Index of genetic and epigenetic diversity in HK was low. The reason could be HK population was located in Hainan Province, which was an independent island, resulting in less frequent gene exchange between HK population with the other populations. As the earliest region in China to introduce *S. trilobata*, GZ has the longest time for the formation of its escape zone and natural community, which may be the reason have led to its highest genetic diversity. During the process of clonal reproduction, plants evolve complex adaptation with defense mechanisms through potential epigenetic traits due to the stress they feel from the environment, and are genetically mediated to produce phenotypic changes called phenotypic plasticity. Studies had shown that invasive species tend to had higher phenotypic plasticity than non-invasive or native species, even that populations of invasive species in invasive regions were able to evolve greater plasticity than native populations [13]. The FZ population had the highest epigenetic diversity, it may be due to the unique distribution of samples in FZ area. Farther distribution between different populations and denser the distribution within the population, would bring on greater epigenetic differentiation between different populations. However, the distribution of samples in other areas was more uniform. At the same time, GZ also has high epigenetic diversity, with only a very slight gap is lower than Fuzhou.

After analyzing the UPGMA phylogenetic tree and PCoA results, it was found that the MSAP had a pronounced effect than the SSR clustering. The epigenetic molecular markers seemed to be able to better distinguish regions, which indicated that epigenetics was more malleable during adaptation to different habitats. It was even more obvious in the PCoA result plot of MSAP that the GZ population was clearly separated from the rest of the region, which was a very interesting phenomenon. As the GZ region was the first to be introduced and cultivated in China, and its population has undergone a long-term stage of adaptive evolution, whereas other regions were introduced and cultivated at a later time. This observation also supports the notion that genetic and epigenetic diversity indices of species increase proportionally with the stage of adaptive evolution.This result was consistent with the findings of Shi et al., indicating that during clonal reproduction, plants evolved complex adaptations with defense mechanisms through underlying epigenetic traits due to environmental pressures, and improved adaptive ability through gene mediation [14]. Moreover, our research reveals a positive trend in the Shannon diversity index of genetic and epigenetic diversity in different populations. Previous studies had shown that incidental genetic variation can greatly shape epigenetic variation in order to truly enrich epigenetic mechanisms interact with genetics to promote heredity and ultimately lead to the transformation of genetic and other non-genetic mechanisms into body performance [31–33]. This also showed that in the invasion mechanism of *S. trilobata*, heredity and epigenetics were mutually supportive.

### Comparison of the different *Sphagneticola trilobata* populations in genetic distances and epigenetic distance with their geographical

Mantel’s analysis revealed that the correlation between epigenetic distance and geographical distance was significantly stronger than that between genetic distance and geographical distance, indicating that epigenetic distance was more affected by geographical distance. Additionally, the Fst results suggested that epigenetics exhibited greater differentiation than genetics. Herrera et al. discovered that the differences in functional traits of *Helleborus foetidus* were related to environment and epigenetic distance [34]. Similarly, Pagel et al. observed a robust correlation between genetic distance and epigenetic distance in *Linum catharticum* from both dry and wet grasslands [35]. These findings provide evidence that environmental factors play a significant role in shaping genetic and epigenetic levels. In this study, we found that the genetic and epigenetic distances in *S. trilobata* just showed a significantly weakly correlated, where the habitats of our four regions were not in extreme opposition. Besides, Ne, H, I and altitude were significantly positively correlated in genetics, as well as PIC, Na, Ne, H, and I in epigenetics were all positively correlated with the highest temperature (Fig. S2).As a consequence, Adaptation pressures from various habitats are driving the genetic and epigenetic diversity in the species. Environmental factors had a certain degree of influence on the genetic and epigenetic levels, with a more significant effect on epigenetics.

### Differences epigenetic composition in *S. trilobata* rapid invasion

The methylation rate in plants typically falls within the range of 20% to 40% [36]. In this study, the average methylation rate of *S. trilobata* in four regions ranged from 18.25%—32.92%. The results manifested that *S. trilobata* was significant differences in methylation among the four populations. The methylated DNA can cause chromatin extremely to become highly compact [37], resulting in reduce genetic exchange and a decrease in polymorphic bands. Furthermore, since type IV (0 0) was an unintuitive amplification site, the number of methylation sites detected by MSAP may be underestimated, implying that the actual level of methylation in *S. trilobata* may be higher than reported. The total methylated rate (%) and fully methylated rate (%) were both the highest in HK population, whereas those in GZ population were the lowest. The hemi-methylated rate (%) was the highest in FZ population. The non-methylated rate was the highest in GZ population and the lowest in HK population. Overall, the methylation level of GZ population was significantly lower than that of other populations, indicating that there were some differences in methylation patterns between different populations. This could be attributed to variations in local temperature and habitats at the sampling sites. In cloned plants, epigenetic modifications can created new modules over time and accumulate them for adaptations [15, 38]. There were abundant evidences suggesting that sexual and clonal reproduction can effectively superimposed, with epigenetic regulation participating in and regulating this clonal reproduction [39, 40].

For example, Shi et al. (2019) discovered a substantial amount of epigenetic differences within the clone population of *Alternanthera philoxeroides* in the same environment, which can be maintained until 10 generations before gradually disappearing, indicating that epigenetic variation was likely caused by environmental induction and spontaneous mutations [14]. Similarly, Guarino et al. (2019) denoted on the invading clonal population *Arundo donax,* which showed that epigenetic diversity was higher than genomic diversity, suggesting its invasion under different environmental conditions was attributed to higher epigenetic variability [41].

The differences in methylation patterns in different regions of *S. trilobata* indicated that the methylation patterns of cloned plants produced significant differences in invasion regions. These results suggest that epigenetic mechanisms partially compensate for the limited gene exchange between populations due to clonal reproduction. This indicates that epigenetics plays a supportive role in the process of adaptation and evolution, and exhibits greater plasticity in aiding clonal plants to adapt to invasion.

## Conclution

As can be concluded from this study, *S. trilobata* populations in different regions had high genetic and epigenetic diversity, which have played a mutually reinforcing role in facilitating its rapid invasion. The underlying drivers for these changes are attributed to diverse environmental pressures, which have been one of the primary factors responsible for *S. trilobata*'s successful invasion. Notably, the correlation between epigenetics and geographic distance was significantly higher than that observed for genetic and geographic distance. By virtue of the significant difference in methylation patterns among different populations, the epigenetic mechanism of *S. trilobata* have endowed it with greater plasticity, thereby enabling it to adapt rapidly to changing environments. Overall, this study lays a foundation for a deeper understanding of how cloned plants undergo rapid adaptation.

## Supplementary Information

**Additional file 1: Table S1.** Components of enzyme digestion system for MSAP (20 μl).** Table S2.** Formula of adapters for MSAP. **Table S3.** System of ligation for MSAP (20 μl). **Table S4.** Reaction system of pre-amplification (20 μl). **Table S5.** Reaction system of selection PCR amplification. **Table S6.** Sequences of adapters and primers for MSAP. **Table S7.** Reaction system of SSR amplification (10μl). **Figure S1.** Sampling sites distribution of Sphagneticola trilobata. **Figure S2.** Correlation between epigenetic (a) and genetic diversity (b) of Sphagneticola trilobata and environmental factors.

## Data Availability

All relevant data can be found within the manuscript and its supporting materials. All data analyzed during this study are included in the supplementary information fles.
