# Supplementary material for: Comparative genetic and epigenetic of the Sphagneticola trilobata (L.) Pruski from different regions in China
Source: BMC Plant Biol. 2023 May 30;23:289. doi: 10.1186/s12870-023-04277-w (PMC10228135; doi:10.1186/s12870-023-04277-w)
Supplement: Supplementary file 1 — Additional file 1: Table S1. Components of enzyme digestion system for MSAP (20 μl). Table S2. Formula of adapters for MSAP. Table S3. System of ligation for MSAP (20 μl). Table S4. Reaction system of pre-amplification (20 μl). Table S5. Reaction system of selection PCR amplification. Table S6. Sequences of adapters and primers for MSAP. Table S7. Reaction system of SSR amplification (10μl). Figure S1. Sampling sites distribution of Sphagneticola trilobata. Figure S2. Correlation between epigenetic (a) and genetic diversity (b) of Sphagneticola trilobata and environmental factors. [file 12870_2023_4277_MOESM1_ESM.pdf]

Method

1.DNA extraction

DNA of each sample was extracted and stored at − 20 °C. DNA extraction were extracted using plant genome DNA extraction kit (Beijing Tiangen).

2.MSAP Method

2.1 Enzyme digestion system

EcoRI combined with two isoschizomers which are differently sensitive to DNA methylation (EcoRI/HpaII and EcoRI/MspI) (all enzymes are purchased from Takara) was incubated at 37 ℃. The optimal incubated time is 8 h (Table S1) (Primer synthesis consists of Sangon biotech).

Table S1 Components of enzyme digestion system for MSAP (20 μl)

Components	Volume(μl)
HapII/MspI（10 U/μl）	1.35
EcoRI（10 U/μl）	1.35
BSA	2.0
10 x T Buffer	2.0
DNA（600 ng/μl）	8.0
ddH_2O	5.3
Total	20.0

2.2 DNA ligase

The DNA fragments were ligated to the adapters. The Formula of adapters were amplified for 95 ℃, 5 min; 65 ℃, 10 min; 37 ℃, 10 min; 25 ℃, 10 min. The mixture was incubated at 16 ℃ overnight, inactivated at 65℃ for 10 min, and stored at −20 ℃ (Table S2;Table S3).

Table S2 Formula of adapters for MSAP

EcoRI adapters（5 μM）	MspI adapters（50 μM）
EcoRI adapters	MspI adapters
10 μl 100 μM EcoRI adaptersI	100 μl 100 μM MspI adaptersI
10 μl 100 μM EcoRI adaptersII	100 μl 100 μM MspI adaptersII
180 μlddH$_2$O	

Table S3 System of ligation for MSAP (20 μl)

Components	Volume
EcoRI adapters(5 μM）	1.0
HapII/MspIadapters（50 μM）	1.0
T4 DNA Ligase	0.4
T4 DNA ligase buffer (10×)	2.0
the DNA fragment	10.0
ddH$_2$O	5.6
Total	20.0

2.3 Pre-amplification

The dna fragments was is diluted 10 times，Conditions of the PCR amplification were as follows: 94°C, 3 min; 20 cycles of 94°C, 30 s; 56°C, 60s; 72°C, 60 s, 72°C, 10 min (Table S4).

Table S4 Reaction system of pre-amplification (20 μl）

Components	Volume(μl)
EcoRI primer(10μM)	1.0
HapII/MspI primer(10μM)	1.0
Taq DNA Polymerase(5U/μl)	0.2
dNTPs（2mM）	2.0
10 x PCR Buffer	2.0
The amplifed fragment	4.0
dd H_2O	9.8

2.4 Selection PCR amplification

Conditions of the PCR amplification were as follows:94 ℃, 5 min;13 cycles of (94℃，30 s;65 ℃, 1 min;72 ℃, 1 min);23 cycles of(94 ℃, 30 s;,56 ℃, 72 ℃, 1 min),The annealing temperature decreases by 0.7 ℃ per cycle, and a final extension at 72 ℃ for 10 min (Table S5, Table S6).

Table S5 Reaction system of selection PCR amplification

Components	Volume(μl)
EcoRI primer(10μM）	1.0
HapII/MspI primer(10μM)	1.0
Taq DNA Polymerase(5U/μl)	0.2
dNTPs(2mM)	2.0
10 x PCR Buffer	2.0
The amplifed fragment	2.0
dd H_2O	11.8
Total	20.0

2.5 Denaturing polyacrylamide gel electrophoresis

Using 6% denaturing polyacrylamide gels, electrophoresis was performed at a constant power of 120V for 2h. After silver staining, a statistical analysis was carried out.

Table S6 Sequences of adapters and primers for MSAP

	**Primers/Adapters**	**Primer sequence (5'-3')**
	Eco-adapterI	CTCGTAGACTGCGTACC
Adapters	Eco-adapterII	AATTGGTACGCAGTCTAC
	H/M-adapterI	GATCATGAGTCCTGCT
	H/M-adapterII	CGAGCAGGACTCATGA
Pre-selective amplification	Pre-EA	GACTGCGTACCAATTCA
	Pre-HM	ATCATGAGTCCTGCTCGGT
	Eco-ACA	GACTGCGTACCAATTCACA
	Eco-ACC	GACTGCGTACCAATTCACC
	Eco-ACCA	GACTGCGTACCAATTCACCA
	Eco-AAC	GACTGCGTACCAATTCAAC
	Eco-ACA	GACTGCGTACCAATTCACA
Selective amplification	Eco-ACT	GACTGCGTACCAATTCACT
	H/M-TAG	ATCATGAGTCCTGCTCGGTAG
	H/M-TCA	ATCATGAGTCCTGCTCGGTCA
	H/M-TAC	ATCATGAGTCCTGCTCGGTAC
	H/M-TCCA	ATCATGAGTCCTGCTCGGTCCA
	H/M-TCAA	ATCATGAGTCCTGCTCGGTCAA
	H/M-TCG	ATCATGAGTCCTGCTCGGTCG

3.SSR Method

3.1 SSR-PCR

Conditions of the PCR amplification were as follows:95℃, 3 min; 34 cycles of (95℃, 30 s; The annealing temperature is the annealing temperature of each primer, 30 s ; 72℃, 60 s); and a final extension at 72 ℃ for 5 min and finally stored at -20 °C(Table S7, Table S8).

Table S7 Reaction system of SSR amplification (10μl)

Components	Volume(μl)
F primer(0.5μL)	0.5
R primer(0.5μL)	0.5
2 × Taq PCR Master Mix 5 μL	5.0
The DNA fragment	1.0
dd H_2O(3μL)	3.0

Table S8 Characteristics of 6 SSR loci developed in *Sphagneticola trilobata*

loci	Repeat motif	Size Range(bp)	Tm(℃)	Primer sequence (5'-3')
P80	$(TG)_{10}$	174-176	60	F: GATGGGTGCGCAATTCTTAT<br>R: GTGGCCTGCTGCTAGTCTTC
P83	$(TG)_{9}$	255-259	60	F: GAAGCATTAGCACCGGAAAG<br>R: TCCCCACTATCAACTCCACC
P119	$(AG)_{5}$	162-168	50	F: GGTGTACTGACCTCGAATA<br>R: CAACCCTCAACAGAAGAA
P123	$(CAA)_{13}$	188-212	49	F: AATCCCTCTTCATTCGTG<br>R: CTGCTGCTGTTGTTGGTG
P124	$(TCA)_{8}$	165-168	50	F: AGCCTGTTTATTCCCATTT<br>R: CTTCTCACGGAGTTTGTAG
P152	$(GT)_{9}$	238-248	60	F: TAACTTCTGGCAAACGGAGG<br>R: CAGTATCAATCGCCTAGCCTG

4. Data analysis

The distribution model of maximum entropy (MaxEnt) was used to predict potential distribution of *S. trilobata* in China under current.The mode usage data were mainly derived from the records of the research group's field investigation of the geographical distribution of *S. trilobata*, mainly focused on Guangdong, Guangxi, Fujian, Yunnan, Hainan and other provinces and regions.

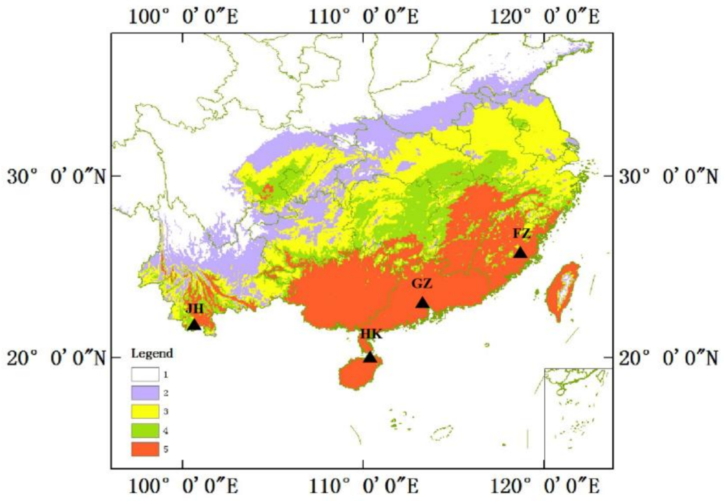

Figure S1 Sampling sites distribution of *Sphagneticola trilobata*

Note: The numeric value represents the level of risk, higher numbers indicate higher risk.

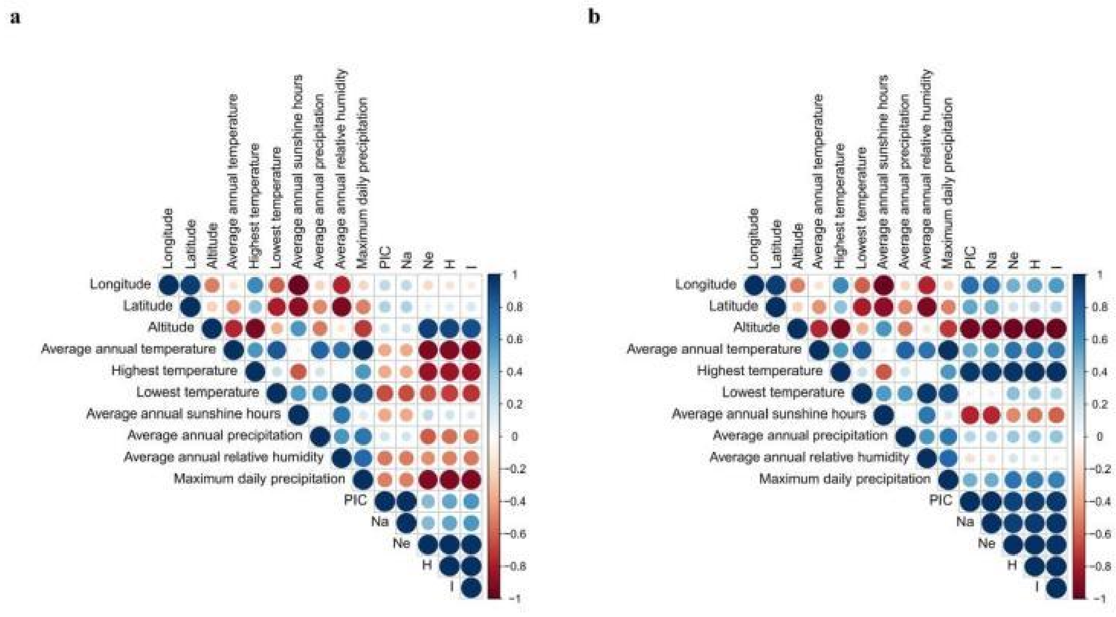

Figure S2 Correlation between epigenetic (a) and genetic diversity (b) of *Sphagneticola trilobata* and environmental factors
